# Microscopic ion migration in solid electrolytes revealed by terahertz time-domain spectroscopy

**DOI:** 10.1038/s41467-019-10501-9

**Published:** 2019-06-17

**Authors:** Tomohide Morimoto, Masaya Nagai, Yosuke Minowa, Masaaki Ashida, Yoichiro Yokotani, Yuji Okuyama, Yukimune Kani

**Affiliations:** 1 Graduate School of Engineering Science, Osaka University, Toyonaka, Osaka 560-8531 Japan; 2 Panasonic Science Research Alliance Laboratories, Graduate School of Engineering, Osaka University, Suita, Osaka 565-0871 Japan; 3 Institute for Transdisciplinary Graduate Degree Programs, Osaka University, Toyonaka, Osaka 560-8531 Japan; 4 Faculty of Engineering, Department of Environmental Robotics, University of Miyazaki, 1-1 Gakuenkibanadai-nishi, Miyazaki, 889-2192 Japan; 5 Technology Innovation Division, Panasonic Corporation, 3-1-1 Yagumo-nakamachi, Moriguchi City, Osaka 570-8501 Japan

**Keywords:** Fuel cells, Terahertz optics

## Abstract

Terahertz spectroscopy is one of the most suitable methods for the analysis of electron transport in solids, and has been applied to various materials. Here, we demonstrate that terahertz spectroscopy is the technique of choice to characterize solid electrolytes. We measure the terahertz conductivity of stabilized zirconia, a widely used solid electrolyte material, by terahertz time-domain spectroscopy at high temperatures, providing a wealth of information unavailable from conventional techniques. It is found that the conductivity reflects the microscopic motion of the ion just before hopping to an unoccupied site. Our results suggest a powerful approach in probing the ionic conduction mechanism and could help us explore other solid electrolytes for fuel cells and all-solid-state batteries.

## Abstract (web summary)

Here the authors expand the application scope of terahertz spectroscopy to the characterization of electrolyte materials. Based on the measurement of the conductivity of yttria-stabilized zirconia in the terahertz frequency range, unprecedented insight into the microscopic motion of the ion is revealed.

## Introduction

**Fig. 1:**
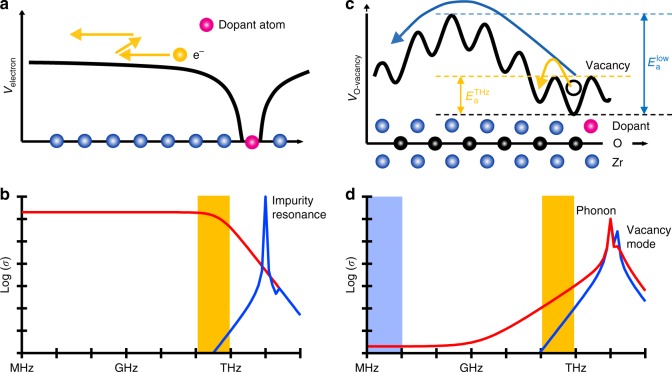
Schematics of electronic and ionic transport. **a** Potential for the electron near a donor atom in a semiconductor. **b** Corresponding conductivity spectra at low (blue) and high temperatures (red). The frequency component of the electronic conductivity at high temperature extends to the THz regime. The shaded area shows the sub-THz frequency region. **c** Potential for the oxygen vacancy near a dopant atom in an oxide ion conductor. **d** Corresponding conductivity spectra at low (blue) and high temperatures (red). The broadening of the vacancy mode towards low frequencies at high temperatures provides information on the individual ion hopping (orange shaded region), which is different from the information obtained in long-range ion transport measurements for low-frequency conductivity (blue shaded region)

Terahertz (THz) conductivity measurements are one of the most powerful and popular tools in present material science to evaluate the carrier transport in solids^1–3^. At low temperatures, the electrons and holes in a doped semiconductor are trapped by the impurity atoms. Here, the resonance frequencies appear in the THz regime (typically several THz). When the carriers are thermally activated at high temperatures, the resulting free electrons and holes exhibit a large conductivity at zero frequency. Since the electronic transport in semiconductors is governed by electron–lattice scattering on the sub-picosecond timescale, the electronic conductivity has also a component in the THz frequency range^4,5^. The schematics of the electron transport and the corresponding conductivity spectra are shown in Fig. 1a, b, respectively. Recent advances in ultrashort laser and frequency conversion technologies have enabled coherent THz wave generation and detection^2,6,7^, which allows us to perform THz time-domain spectroscopy (THz-TDS) measurements with high spatio-temporal resolution without need of electrodes. THz conductivity measurements have been performed on various electronic materials including strong correlated electron systems^1,4,8^.

We propose THz-TDS as an advanced method for the characterization of ion transport in solid electrolytes, which is decisive for the performance of fuel cells or all-solid-state battery^9–11^. Fuel cells have been getting enormous attentions as a new energy generator, because they have various advantages such as high efficiency and cleanliness, compared to conventional power sources^12,13^. All-solid-state battery also has much benefits, such as higher charging speed and higher stability than the conventional batteries^14^. Here, we picked up the stabilized zirconia as an example of solid electrolyte. The stabilized zirconia is the one of the most popular and familiar solid electrolytes and is widely used in commercial solid oxide fuel cells (SOFCs)^15,16^. The ion conduction is usually interpreted in terms of hopping as shown in Fig. 1c. Acceptor dopants located at the cation sublattice in the crystal generate oxygen vacancies, and an adjacent oxygen can hop to an unoccupied site at adequately high temperatures. The energy of this potential barrier is also termed migration energy, which is one of the most important parameters for the intrinsic ion conduction^17^. However, even at the operating temperature of the SOFCs, some oxygen vacancies form defect clusters and stay at specific sites near the acceptor dopants^18^. The stability of the defect cluster is expressed with the binding energy, which strongly modulates the shape of the long-range potential of the acceptor ion. The long-range potential (also called activation energy $E_{\mathrm{a}}^{{\mathrm{low}}}$ indicated with the blue dashed line in Fig. 1c) is the sum of migration energy and binding energy, and can be evaluated using conventional low-frequency (<MHz) impedance measurements. The THz-TDS allows us to investigate the microscopic potential in the oxide ion conductor (that is, the potential that is involved in the hopping process indicated with the orange arrow in Fig. 1c), which is crucial for SOFC material design.

We believe the motion of the ions in the THz frequency range reflects the microscopic ion hopping. Oxygen ions near the vacancy oscillate thermally at the so-called attempt frequency (several THz) before hopping occurs. Such an ion motion can be regarded as a single vacancy mode in accordance with the picture of the hole in an electronic system. It is similar to the localized ion mode in a semiconductor^19^, which is different from the collective ion motion (phonon)^1,20–22^. Since the vacancy modes have strong anharmonicity^23^, a vacancy oscillation with a large amplitude at high temperature induces the hopping to adjacent sites. The frequency components slightly below 1 THz in the conductivity spectra become apparent at high temperatures when the amplitude of the localized vacancy mode is large^24–26^. The expected conductivity spectra at low and high temperatures are shown in Fig. 1d. Therefore, the THz conductivity in a solid electrolyte contains essential information on the individual ion hopping, which is strongly different from the information provided by the long-range low-frequency conductivity measurements.

Here, in this work, we perform THz-TDS for stabilized zirconia at high temperatures and show that the evaluated conductivity includes the information on the microscopic motion of the ion just before hopping to an unoccupied site occurs, which cannot easily be evaluated by conventional techniques.

## Results

### THz conductivity measurements

**Fig. 2:**
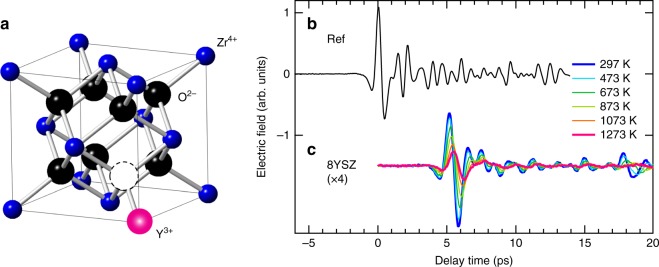
THz time-domain spectroscopy for an 8YSZ pellet. **a** Crystal structure of 8YSZ. **b** The time profile of the reference THz pulse that is obtained without sample. **c** The time profiles of the THz pulses that have passed through the 370 μm-thick 8YSZ pellet at different temperatures. This data set is offset for clarity

We employ THz-TDS to investigate the conductivity in yttria-stabilized zirconia Zr_0.84_Y_0.16_O_2−δ_ (8YSZ), which is commercially used as SOFC electrolyte with the cubic fluorite structure^15,27^ shown in Fig. 2a. Pure ZrO_2_ undergoes a phase transition from the monoclinic structure to the tetragonal structure at a temperature *T* of about 1400 K, and transits to the cubic structure at ~2570 K^27,28^. The doping with 8 mol% Y_2_O_3_ enables stabilization of the zirconia’s cubic fluorite structure even at room temperature^27^ and simultaneously creates oxygen vacancies. Therefore, the oxygen ions can move to the vacancy site, which constitutes the ion conduction. The ion conduction in 8YSZ has been investigated by conventional impedance measurements^29–37^, and 8YSZ is actually utilized as solid electrolyte in oximeters and fuel cells^15^. The ion transport in 8YSZ has also been discussed theoretically^17,38^. Furthermore, the phonon modes have been determined by neutron scattering^39,40^ and Fourier infrared spectroscopy^41,42^.

Figure 2b shows the time profile of the reference THz pulse that is detected without the sample, and Fig. 2c shows the THz waves that are detected after the reference pulses have passed through the 370 μm-thick ceramic 8YSZ plate at different temperatures. The details of our experimental setup for the THz-TDS are described in the Methods. The amplitude of the THz pulse that is transmitted through the zirconia sample at room temperature (Fig. 2b; blue curve) is smaller than that of the incident (reference) pulse and also exhibits a retardation of 6 ps. The amplitude reduction is a result of the reflection and absorption by the sample and the time delay reflects the slow phase velocity of the THz pulse in 8YSZ^22^. We find that, as the sample’s temperature is increased to 1273 K the amplitude decreases further. This suggests that the absorption or conductivity in 8YSZ changes at higher temperatures.

**Fig. 3:**
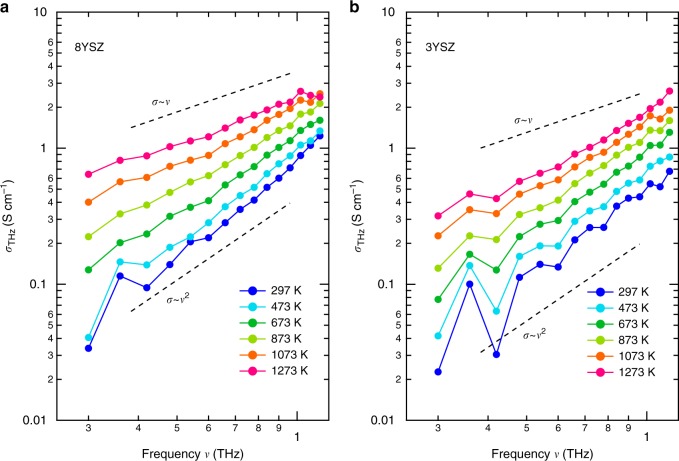
THz conductivities of 8YSZ and 3YSZ. **a** The real parts of the optical conductivities of 8YSZ and **b** those of 3YSZ at different temperatures. The dashed lines represent the linear and quadratic dependences for reference

In order to perform a quantitative analysis of the change in the conductive properties, we evaluate the THz conductivity σ_THz_ from Fourier-transformed electric field profiles of the detected THz pulses (see Methods). Figure 3a plots the real part of the THz conductivity in 8YSZ at different temperatures under ambient atmosphere on a double-logarithmic scale. The real part of the THz conductivity and the real part of the dielectric constant are shown on a linear scale in Supplementary Fig. 1a and 1b, respectively. At room temperature, the THz conductivity increases with the frequency *v*. Additionally, also an increase in the temperature leads to an increase of the THz conductivity, in particular, at the lower frequency region and hence the slopes of the spectra decrease at higher temperatures. Using a single crystal, we verified that these trends hardly depend on the crystallinity (see Supplementary Fig. 2; the THz conductivity of an 8YSZ single crystal at various temperatures exhibits the same spectral shapes as that of the ceramic sample discussed here).

The origin of the THz conductivity component at high temperatures is important for the physics of the SOFC electrolyte. One may consider that this conductivity component is a result of the spectral broadening of the high-frequency phonon mode. Actually, one infrared-active transverse optical (TO) phonon mode at 7.7 THz has been theoretically predicted for the cubic zirconia crystal^28^. Its spectral width should be proportional to the temperature via inter-phonon scattering^22,37^ while maintaining ${\mathrm{\sigma }} \propto {\mathrm{\nu }}^2$ at the frequency region below the resonance frequency. The THz conductivity spectrum at room temperature is indeed proportional to *v*^2^ (Fig. 2a). However, the slope of the spectrum decreases with temperature, and approaches ${\mathrm{\sigma }} \propto \nu$ at high temperatures. This suggests that the spectral change is not due to the spectral broadening of the phonon.

To confirm the minor contribution of the phonon to the THz conductivity at high temperatures, we measure the THz conductivity of another zirconia sample with a Y_2_O_3_ doping concentration of 3 mol% (hereafter referred to as 3YSZ). It is known that the 3YSZ has an additional phonon resonance at 5 THz due to the tetragonal structure and exhibits a weak ion conduction compared to 8YSZ^15,43^. Figure 3b plots the real part of the THz conductivity in 3YSZ at various temperatures on a double-logarithmic scale. The slope of σ in the 3YSZ sample remained proportional to *v*^2^ even at *T* = 1273 K. This also supports that the spectral change of the THz conductivity in 8YSZ is not governed by the phonon resonance.

### Evaluation of the activation energy

**Fig. 4:**
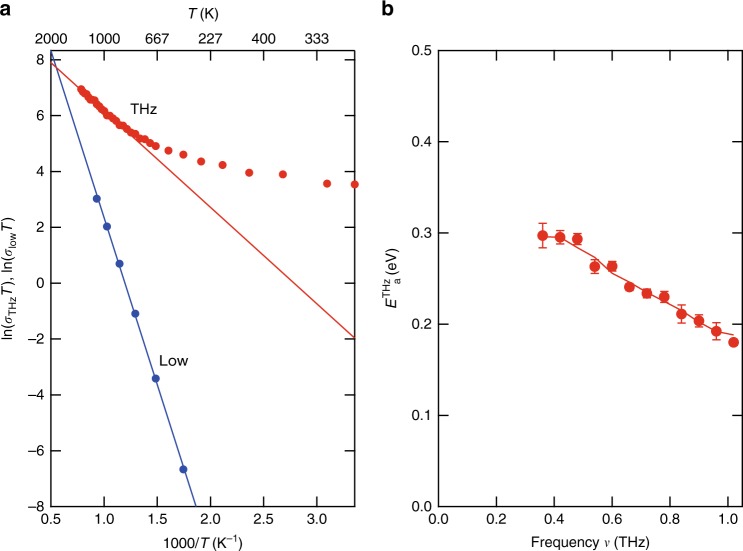
Temperature dependence of the THz conductivity in 8YSZ. **a** Red closed circles represent the temperature dependence of the THz conductivity in 8YSZ at 0.36 THz. Blue closed circles show the conventionally measured low-frequency conductivity in 8YSZ. The solid lines are the fitting results obtained using the Arrhenius equation. **b** The data points show the activation energy evaluated from the THz conductivity of 8YSZ at different frequencies. The solid smoothing curve is a guide to the eye

Another plausible mechanism for the conductivity increase observed in Fig. 3a is the long-range ion conduction that expands toward the THz frequency region. Ion conduction in SOFC electrolytes is explained with the hopping of some oxygen ions to unoccupied sites in the lattice. Various long-range conduction models, such as the correlated barrier hopping model, have been proposed for the frequency region below GHz. These models predict a power law for the high-frequency conductivity that is similar to our results (*σ* = *σ*_0_ + *σ*^*s*^)^35^. Similar discussions have been performed for glass systems^44^. The low-frequency ion conductivity *σ* can be analyzed with the Arrhenius model using

1 $$\sigma = (A/T){\mathrm{exp}}[ - E_a/k_BT]$$

where *A, k*_*B*_, and *E*_*a*_ correspond to the pre-exponential factor, the Boltzmann coefficient, and the activation energy, respectively. The red closed circles in Fig. 4a present the THz conductivity at 0.36 THz. Here, the horizontal axis expresses the inverse temperature and the vertical axis plots ln(σ*T*). Since Eq. (1) can be rewritten as $\ln \left( {\sigma T} \right) = - (E_a/k_B)(1/T) + {\mathrm{ln}}A$, a straight line in this plot suggests that the conductivity obeys the Arrhenius model at high temperatures. In the low-temperature regime, the measured THz conductivity does not follow the straight line due to the phonon contribution as mentioned above. However, as elaborated in the next Section, for the present purpose it is sufficient to fit the high-temperature range. From the slope at high temperatures, we can estimate the activation energy at *ν* = 0.36 THz, $E_a^{THz}$(0.36 THz) = 0.3 eV. Furthermore, Fig. 4b evidences that the activation energy decreases as the THz frequency increases. We also show those for the 8YSZ single crystal and 3YSZ sample in Supplementary Fig. 3, and the prominent features are the same.

### Low-frequency impedance measurements

This activation energy evaluated from the THz conductivity is different from the results obtained by conventional low-frequency impedance measurements. We performed impedance measurements on an 8YSZ ceramic sample and show the Arrhenius plot of the low-frequency conductivity as the blue circles in Fig. 4a. Obviously, the slope of the low-frequency conductivity is larger than that of the THz conductivity. We evaluated an activation energy of $E_a^{low}$ = 1.03 eV, which is consistent with the 0.8–1.1 eV reported previously^31,33–35^. Therefore, the origin of the activation energy obtained by the THz conductivity measurement is different from that obtained by the low-frequency impedance measurements.

## Discussion

As elaborated further below, a vacancy oscillates at a specific site of the lattice until it jumps to an adjacent site. The latter process occurs at a timescale of nanoseconds or longer. This hopping mechanism suggests that the THz conductivity component is related to the large-amplitude vacancy modes, which are relevant for the vacancy hopping. These vacancies modulate phonons. While one TO phonon in cubic zirconia without dopant has been predicted at 7.7 THz^28^, infrared reflection spectroscopy implies a TO phonon mode at 10 to 12 THz in YSZ^41,42^. We also experimentally confirmed this resonance (at 11 THz, as shown in Supplementary Fig. 4). On the other hand, Shin and Ishigame et al. have revealed defect-induced modes around 9 and 21 THz by employing hyper Raman scattering spectroscopy^45^. These modes are infrared-active T_1u_ modes, and have been assigned to the oxygen defect with the analysis of the O_6_ molecule^45^. Thus, we analyze the infrared-active component with the assumption of two contributions, the phonon component with low-nonlinearity and the oxygen vacancy component. Since the potentials for the vacancy modes exhibit a strong anharmonicity^17,38^, the vacancy jumps to the adjacent sites via large-amplitude modes after many attempts. Hence the conductivity spectrum of the vacancy mode extends to low frequencies, and the activation energy at *v* = 0 Hz extrapolated from the THz conductivity includes the information of the microscopic ion hopping.

Let us verify the THz conductivity originating from this vacancy motion by using the conventional analytical technique of the THz vibrational modes in an anharmonic potential^25,26^. While the actual shape of the potential is very complex^17,38^, we approximate the individual potential at each site as Morse potential, which is one of the most typical anharmonic potentials^46^. The energy level *E*_*i*_ of the *i*-th vibrational mode in the Morse potential can be described as $E_i = \left( {i + 1/2} \right)h\nu _0(1 - \chi (i + 1/2))$ in the lowest-order approximation, leading to the following transition energy between the *i*-th level and *i*+1-th level: $E_{i,i + 1} = h\nu _{i,i + 1} = h\nu _0(1 - 2\chi (i + 1))$. Here, *v*_0_ represents the frequency of the lowest vibrational level (*i.e*., the attempt frequency), and *χ* is the anharmonic coefficient^46^. In this model, the depth of the potential *U* is $h\nu _0/4\chi$, and the quantum number is limited by $i < 1/2\chi - 1$, which can be derived from the condition ${\mathrm{\nu }}_{{\mathrm{i}},{\mathrm{i}} + 1} > 0$. By assuming that the population of the vibrational mode, *N*_*i,i*+1_, obeys the Maxwell–Boltzmann distribution, the optical conductivity spectrum (which is directly proportional to the absorption) at the frequency *v* can be written as follows

$$\sigma \propto \nu \mathop {\sum }\limits_{i = 0} \left\{ {\mu _{i,i + 1}^2\left( {N_i - N_{i + 1}} \right)\gamma \left( {\nu - \nu _{i,i + 1}} \right)} \right\}$$

2 $$\propto \frac{{\nu \mathop {\sum }\nolimits_{i = 0} \left\{ {\left( {i + 1} \right)\left( {e^{ - \frac{{E_i}}{{k_BT}}} - e^{ - \frac{{E_{i + 1}}}{{k_BT}}}} \right)\gamma \left( {\nu - \nu _{i,i + 1}} \right)} \right\}}}{{\mathop {\sum }\nolimits_{i = 0} e^{ - \frac{{E_i}}{{k_BT}}}}},$$

where $\mu _{i,i + 1} \propto \sqrt {i + 1}$ represents the transition dipole moment and ${\mathrm{\gamma }}({\mathrm{\nu }} - \nu _{i,i + 1})$ the line width of the *i*-th transition. If we assume that the conductivity for ${\mathrm{\nu }} < 1\,{\mathrm{THz}}$ is mainly governed by a single transition between a certain *i*-th level and the *i*+1-th level, Eq. (2) can be simplified as

3 $$\sigma \propto \nu _{i + 1,i}\left( {N_{i + 1} - N_i} \right) \approx \left( {\frac{1}{{hk_BT}}} \right)e^{ - \frac{{E_i}}{{k_BT}}}$$

in the low-frequency limit of (*E*_*i*+1_–*E*_*i*_)/*k*_*B*_*T*≪1. This relation is equivalent to equation (1), which was used to determine the THz activation energy from the Arrhenius plot (Fig. 4b). Therefore, the activation energy $E_a^{THz}$ (0.36 THz) = 0.3 eV corresponds to the energy *E*_*i*_. Since the sum of zero-point vibration energy ($h{\mathrm{\nu }}_0/2 = 0.02\,{\mathrm{eV}}$ with the assumption of $\nu _0 = 11\,{\mathrm{THz}}$) and *E*_*i*_ in the low-frequency limit is close to the potential barrier *U* for ion hopping, we find *U* ≈ 0.32 eV.

**Fig. 5:**
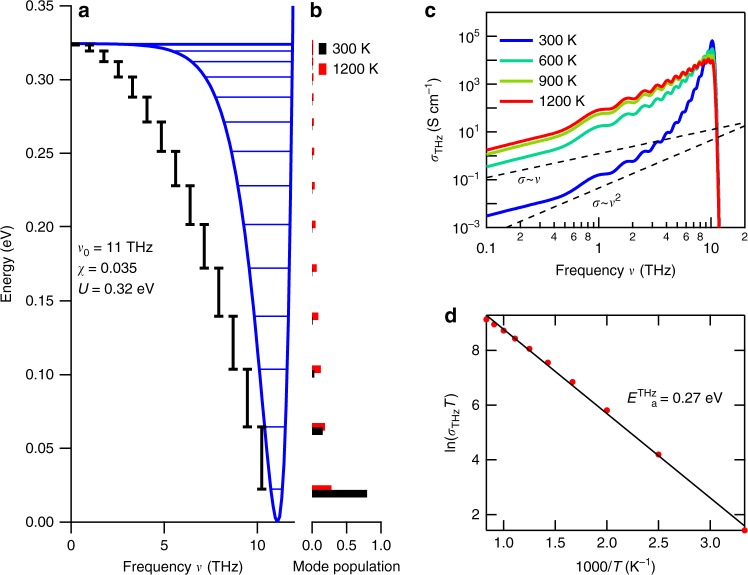
THz ion motions in the Morse potential. **a** The simplified potential shape and energy levels for the localized vacancy mode with ${\mathrm{\nu }}_0 = 11\,{\mathrm{THz}}$ and ${\mathrm{\chi }} = 0.035$. **b** The populations of the energy levels are shown in black and red for 300 and 1200 K, respectively. **c** The calculated conductivity spectra for the localized vacancy mode. **d** The Arrhenius plot for the calculated THz conductivity at 0.36 THz

To clarify whether the above assumptions are valid for this material or not, we simulate the spectra of the THz conductivity using the model outlined above. We set the potential depth to *U* = 0.32 eV, use *v*_0_ = 11 THz as experimentally estimated, and assume *χ* = 0.035 using the relationship $U = h\nu _0/4\chi$. Figure 5a shows the energy diagram of the Morse potential, and Fig. 5b shows the mode populations at 300 and 1200 K considering the Maxwell–Boltzmann distribution. We can derive the THz conductivity from the mode population using Eq. (2), and the results are shown in Fig. 5c. In the THz regime, the components of the conductivity of the localized vacancy mode increase with the temperature, and the conductivity is proportional to the frequency below 1 THz. Figure 5d is the Arrhenius plot of the calculated THz conductivity at 0.36 THz. Indeed, this figure shows a linear relation with an activation energy close to the experimental value $E_a^{THz}$ (0.36 THz) = 0.27 eV. We also simulated the spectra of the THz conductivity using different parameters while maintaining the potential depth. The evaluated activation energy is slightly smaller than the depth of the potential due to the temperature-dependent normalization factor $\mathop {\sum }\limits_{i = 0} \exp \left[ { - E_i/k_BT} \right]$ in Eq. (2). In spite of the simple potential shape, the numerical simulation reproduces the experimental results.

Next, let us specify the site whose potential barrier is reflected in the THz conductivity measurements. When the vacancy moves to a neighboring site, it passes the saddle point of the potential that lies between two cations^30^. An oxygen vacancy at high temperatures remains at the lowest energy site for a long time because even the thermal energy of the vacancy mode at 1000 K is significantly smaller than the energy scale of the binding energy (>0.3 eV^18,47^). This fact suggests that the activation energy $E_a^{THz}$ evaluated from the THz conductivity reflects the minimum potential barrier for the oxygen vacancy at the lowest energy site (indicated with the orange dashed line in Fig. 1c). We note that the expected potential barrier near the dopants is larger than the intrinsic migration energy due to the additional binding energy^23,48^. However, it has been reported that the lowest energy site of the oxygen vacancy is the second-nearest neighbor of the dopant in case of an impurity with a large ionic radius^18,47^. In this case, there are several equivalent sites (located like a shell around the impurity) with the same minimum energy and the vacancy can move to a neighboring site with the same energy via several paths. The potential barrier at the Zr–Zr saddle point^17^ is the minimum energy required for the hopping to the next equivalent site. Therefore, we consider that activation energy determined by the THz conductivity measurements reflects the intrinsic migration energy of the oxygen vacancy.

**Fig. 6:**
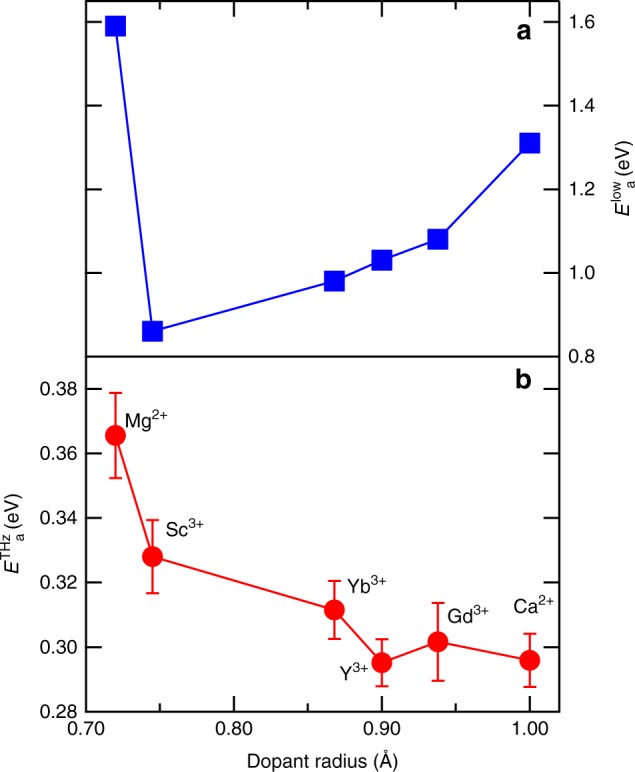
The activation energies for stabilized zirconia samples with different dopant ions. **a** The low-frequency activation energy obtained by impedance measurements. **b** The activation energy in the THz regime obtained by THz-TDS

The abovementioned migration and binding energies, which are relevant for microscopic ion conduction, have been investigated extensively, but the exact values are still a matter of debate in theoretical studies^30,48^. Both parameters have been characterized experimentally using the temperature-dependent slopes of the Arrhenius plots for the low-frequency conductivity^49,50^. However, the evaluated values are likely to exhibit a large uncertainty due to a simplified analysis, leading to unresolved issues even in zirconia^16^. The mechanisms of oxide ion migration (e.g. the formation of oxygen vacancies and their preferential locations) have also been investigated by nuclear magnetic resonance^51^, and muon measurements^52^. However, these are not standard techniques that are easily accessible for a wide range of researchers. Here, we applied a standard technique based on ultrashort optical pulse technology and directly evaluated the oxide ion dynamics in the solid electrolyte of SOFC. This method enables the direct evaluation of the charge fluctuation owning to its high temporal resolution. Our proposed method is very attractive for the field of ionics, because the intrinsic migration energy of the oxygen vacancy can be accurately measured. We measured the THz conductivity for different stabilized zirconia samples to investigate the dependence of the activation energy on the impurity atom type (*M*_2_O_3_ with *M* = Y, Yb, Gd, Sc, and *M*O_2_ with *M* = Ca, Mg). Figure 6a shows the activation energies evaluated by the low-frequency impedance method, $E_a^{low}$. The temperature dependence of the low-frequency impedance is shown in Supplementary Fig. 5. Figure 6b shows the activation energies evaluated by the THz conductivity measurements at ${\mathrm{\nu }} = 0.36\,{\mathrm{THz}}$, $E_a^{THz}$. The graph clearly shows that the THz activation energy decreases with the ionic radius of the dopant, which is different from the result obtained in the impedance measurements. This is because the activation energy obtained in the impedance measurement reflects the long-range potential while the THz activation energy reflects the short-range potential. The trend of the THz activation energy is a result of the increase in the effective lattice constant due to the larger ionic radius of the dopant. Therefore, the migration energy along the Zr–Zr path decreases^17,48^. This tendency is in good agreement with the calculation result^17^.

In conclusion, we measured the THz conductivity of various stabilized zirconia samples by THz-TDS. We observed the THz responses originating from the large-amplitude oxygen-vacancy motion, which is related to the vacancy hopping to adjacent sites. The temperature dependence of the THz conductivity revealed the intrinsic migration energy of the vacancy. This method is applicable to various solid electrolytes in protonic ceramic fuel cells (PCFC)^53–57^ and all-solid-state lithium ion batteries^10,58^, because the attempt frequencies of the ion transport in these materials are also expected to lie around several THz^59^. Since THz-TDS should reveal the hidden mechanism of ionic conduction, our proposed method is useful for development of novel solid electrolytes for fuel cells or all-solid-state batteries.

## Methods

### Sample preparation

The Zr_0.84_Y_0.16_O_2-δ_ and Zr_0.88_Sc_0.12_O_2-δ_ pellets were prepared using commercial powder. The Zr_0.84_Y_0.16_O_2-δ_ powder (TOSOH) and the Zr_0.88_Sc_0.12_O_2-δ_ powder (DAIICHI KIGENSO KAGAKU KOGYO) were first pressed into pellets at 250 MPa and then sintered at 1673 K for 5 and 2 h in air, respectively. The Zr_0.84_*M*_0.16_O_2-δ_ (*M*=Mg, Yb, Ca) samples were prepared by a solid-state reaction method (SSR). The reagent-grade ZrO_2_ (99.9 %), MgO(99.9%), Yb_2_O_3_ (99.9 %), and CaCO_3_ (99.9 %) powders were weighed and mixed in ethanol using a mortar and a pestle. The powder mixtures were calcined in air for 5 h at 1273 K. The calcined Zr_0.84_*M*_0.16_O_2-δ_ (*M* = Mg, Yb, Ca) powders were ball-milled and pressed into pellets at 250 MPa and then sintered at 1873 K for 5 h in air. The Zr_0.84_Gd_0.16_O_2-δ_ pellets with single phase were prepared by a combustion synthesis method. ZrO(NO_3_)_3_ 2.1H_2_O(99.9 %), Gd(NO_3_)_3_ 6.1H_2_O (99.9 %), water, citric acid, and ethylenediamine tetraacetice acid were mixed while maintaining a pH of 10 by adding aqueous ammonia. After ammonium nitrate was added, the solution was heated at 623 K. The obtained powders were calcined in air for 10 h at 1173 K. The powder was further ball-milled and pressed into CIP pellets at 250 MPa, which was followed by sintering at 1873 K for 10 h in air. The obtained samples were used for both the THz conductivity measurements and the impedance measurements. For characterization, we measured the X-ray diffraction (XRD) of the powder samples using MinFlex600 (Rigaku) with CuKα radiation. All XRD patterns shown in Supplementary Fig. 6 were analyzed by employing the Rietveld refinement technique. The X-ray powder diffraction analysis confirmed a well-defined fluorite pattern for all samples prepared in this study.

### Experimental setup for the THz time-domain spectroscopy

We performed the THz-TDS measurements by using the output from an Er-doped fiber laser (TOPTICA Photonics, FemtoFiber pro NIR) with a center wavelength of 780 nm (second harmonic of 1560 nm), a repetition rate of 80 MHz, an average power of 130 mW, and a pulse duration of 90 fs. A beam splitter was used to divide the output beam into the excitation beam for THz pulse generation and the sampling beam for detection, respectively. The excitation pulse was focused on an InAs plate exposed to a magnetic field of 0.5 T. The THz pulses emitted from InAs were guided by four parabolic mirrors to the sample and transmitted to an electro-optic (EO) detector, which was a 1-mm-thick (110)-oriented ZnTe crystal. The instantaneous THz electric field amplitude alters the birefringence of the ZnTe crystal. The sampling pulses were focused on the same crystal, and the polarization state of the sampling pulse modulated by the THz wave was measured using a quarter wave plate, a Wolston prism, and balanced Si detectors. To achieve a high signal-to-noise ratio in the measurements, the excitation pulses were chopped at 6 kHz and the modulated signal was analyzed with a lock-in amplifier. The sample was placed in an electric furnace with temperatures up to 1300 K under atmosphere without dry-gas replacement, and the THz pulse entered the furnace through an aperture with 7 mm diameter (no window) using an off-axis parabolic mirror. This setup has a low-frequency detection limit of 0.35 THz. The black-body radiation from the sample can hardly influence our measurements because its field amplitude is much lower than that of the coherent THz pulse. Nevertheless, we carefully blocked the black-body radiation from the electric furnace with thin black polypropylene films.

### Evaluation of complex optical conductivity

Using THz-TDS, we determined the electric field waveform of the THz pulse transmitted through the sample, $E_s(t)$, and that of the reference pulse without sample, $E_r\left( t \right)$. The complex optical conductivity $\tilde \sigma (\omega )$ can directly be evaluated with these time profiles as explained in the following. First, we only employ the measured electric field waveforms in the time region from 2 to 16 ps, because the signal at 18 ps caused by multiple reflections in the sample complicates the analysis. Second, the complex transmittance $\tilde t\left( \omega \right)$, which is a ratio between the Fourier-transformed electric fields $\tilde E_s\left( \omega \right)$ and $\tilde E_r\left( \omega \right)$, can simply be expressed in terms of the complex permittivity $\tilde \varepsilon (\omega )$,

4 $$\tilde t\left( \omega \right) = \frac{{\tilde E_s\left( \omega \right)}}{{\tilde E_r\left( \omega \right)}} = \frac{{4\sqrt {\tilde \varepsilon (\omega )} }}{{\left( {\sqrt {\tilde \varepsilon (\omega )} + 1} \right)^2}}\exp \left[ {\frac{{i\left( {\sqrt {\tilde \varepsilon (\omega )} - 1} \right)d\omega }}{c}} \right]$$

Here, *c* is the speed of light (in vacuum) and *d* is the sample thickness. Thus, we can obtain $\tilde \varepsilon (\omega )$ from $\tilde t\left( \omega \right)$ and then evaluate the complex conductivity $\tilde \sigma (\omega )$ using the following relation,

5 $$\tilde \varepsilon \left( {\mathrm{\omega }} \right) = \varepsilon _\infty + \frac{{\tilde \sigma \left( \omega \right)}}{{i\omega }}$$

### Impedance analysis

The electrical conductivities of the samples were measured by a two-probe alternating-current technique. The samples prepared for this measurement had the shape of a pellet (length: 0.5–1.0 mm). The Pt electrodes were prepared by applying a Pt paste on both surfaces of the pellet and subsequent baking at 1173 K in air. The samples were equilibrated with 1.9% H_2_O–21% O_2_–Ar in the temperature range of 573–1073 K. The complex impedance was measured in the frequency range of 4–8 MHz using an inductance–capacitance–resistance meter (HIOKI corporation: IM3536).

## Supplementary information

Supplementary Infomation

## Data Availability

All data needed to evaluate the conclusions in the paper are present in the paper and/or its Supplementary Information. All other data are available from the corresponding author upon reasonable request.
