# Supplementary material for: Microscopic ion migration in solid electrolytes revealed by terahertz time-domain spectroscopy
Source: Nat Commun. 2019 Jun 17;10:2662. doi: 10.1038/s41467-019-10501-9 (PMC6572842; doi:10.1038/s41467-019-10501-9)
Supplement: Supplementary file 1 — Supplementary Infomation [file 41467_2019_10501_MOESM1_ESM.pdf]

Supplementary Information

Microscopic ion migration in solid electrolytes revealed by terahertz time-domain spectroscopy

T. Morimoto et al.

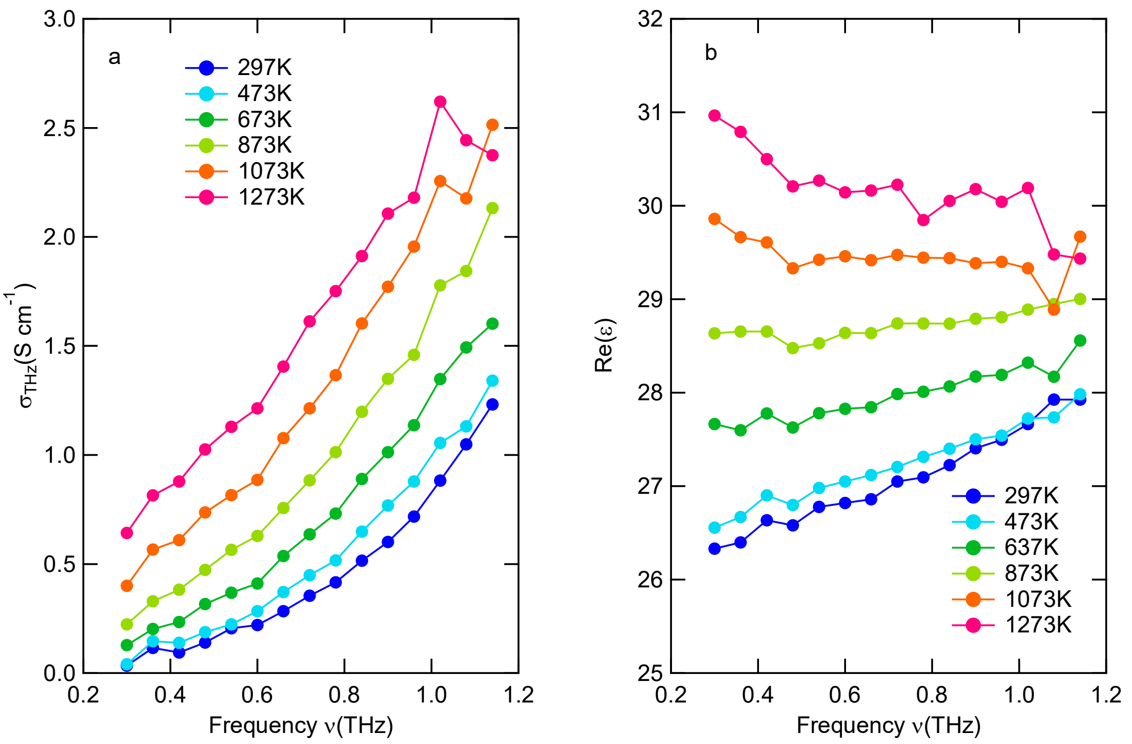

Supplementary Figure 1| The real part of the conductivity (a) and the dielectric constant (b) in the THz frequency regime for the 8YSZ pellet.

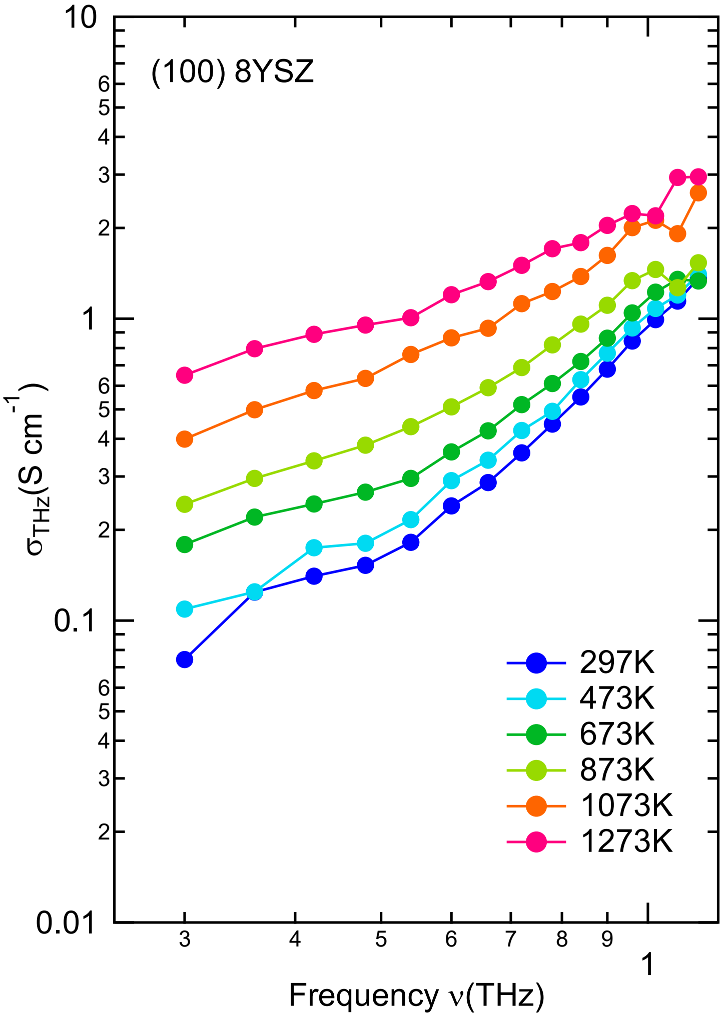

Supplementary Figure 2| THz conductivity of an 8YSZ single crystal. Real part of the THz conductivity of an 8YSZ single crystal (Shanghai Daheng Optics& Fine Mechanics Co.) at various temperatures. The single crystal's size, thickness, and orientation are 10×10 mm, 0.5 mm, and (100). The sample was grown with the arc-zone melting method.

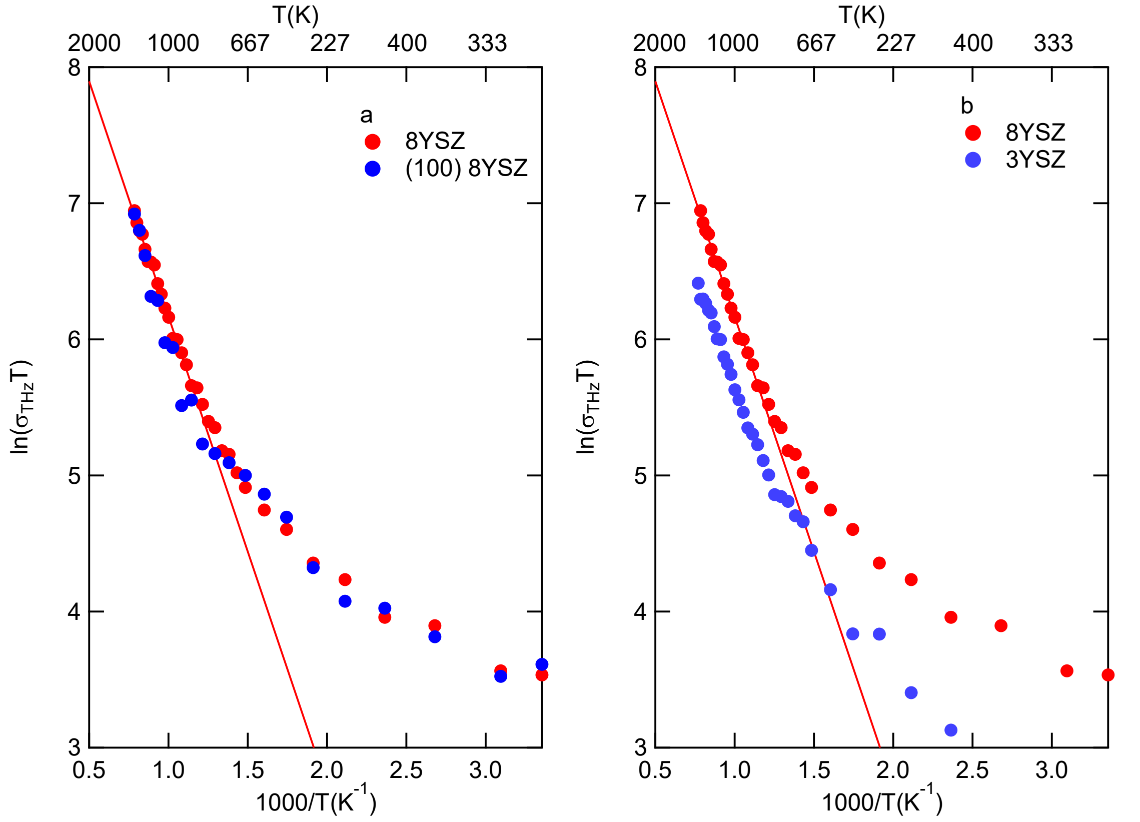

Supplementary Figure 3| The temperature dependence of the THz conductivity in an 8YSZ single crystal and 3YSZ. a, The Arrhenius plot of the THz conductivity of the ceramic pellet (red closed circles) and the single crystal (blue closed circles) at 0.36 THz. **b,** The Arrhenius plot of the THz conductivity in 3YSZ (blue closed circles) and 8YSZ (red closed circles) at 0.36 THz. The solid line is the fitting curve shown in Fig. 4a.

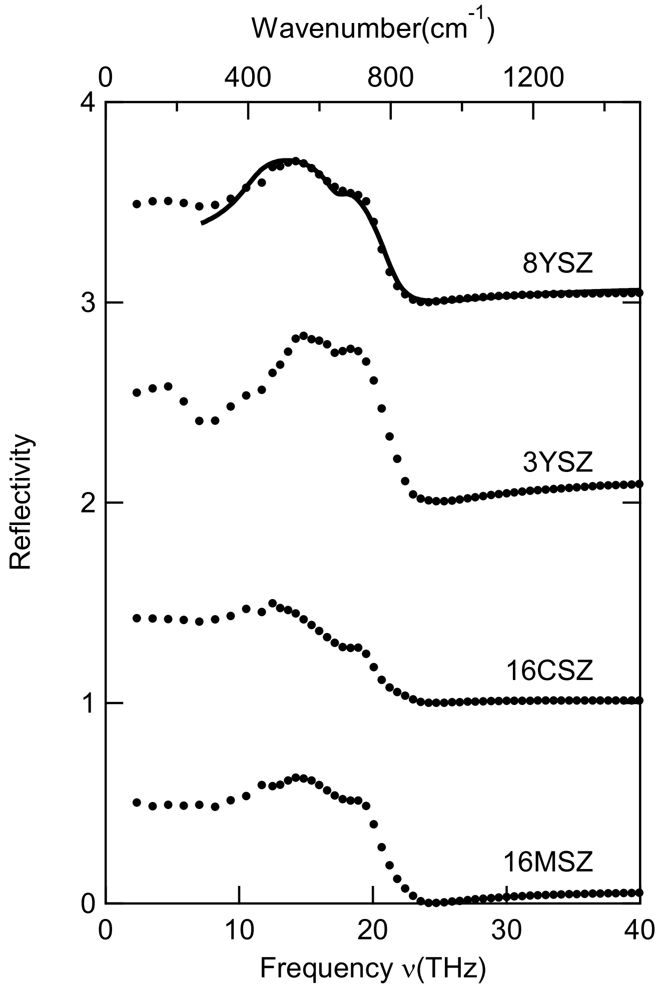

Supplementary Figure 4| Infrared reflection spectra of three different stabilized zirconia samples. Infrared reflection spectra of $Zr_{0.84}M_{0.16}O_{2-\delta}$ (M= Y, Ca, Mg) and $Zr_{0.94}Y_{0.06}O_{2-\delta}$ as measured by Fourier-transformed infrared spectroscopy. The solid line is the fitting curve of 8YSZ obtained using a Lorentz model with two oscillators: $\varepsilon(\omega) = \varepsilon_\infty + \sum_i \left[S_i / \left(\omega_i^2 - \omega - i\omega\gamma_i\right)\right]$. The evaluated fitting parameters are $\omega_1 = 11.1, \gamma_1 = 2.67, S_1 = 1158, \omega_2 = 17.5, \gamma_2 = 2, S_2 = 20$ and $\varepsilon_\infty = 3.5$. We can confirm that the main TO phonon mode is located at 11 THz. Though the reflection spectrum of 3YSZ indicates an additional phonon resonance of 5 THz due to the tetragonal structure, this phonon hardly affects the THz conductivity spectra shown in Fig. 3b.

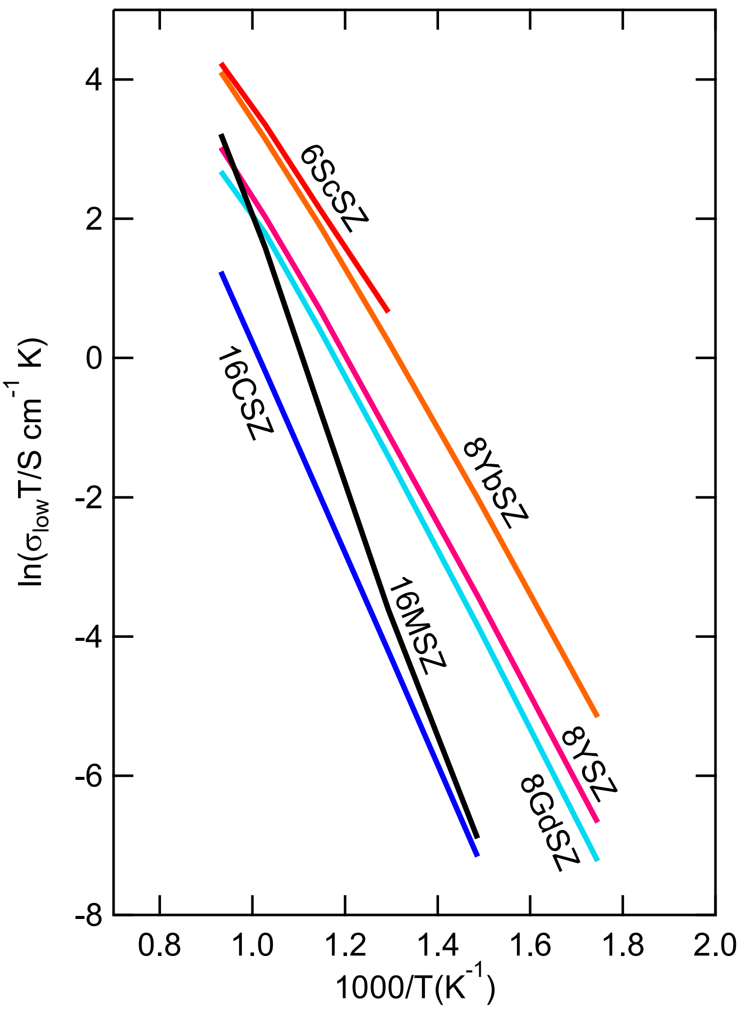

Supplementary Figure 5| The Arrhenius plots for the electrical conductivity in several stabilized zirconia samples.

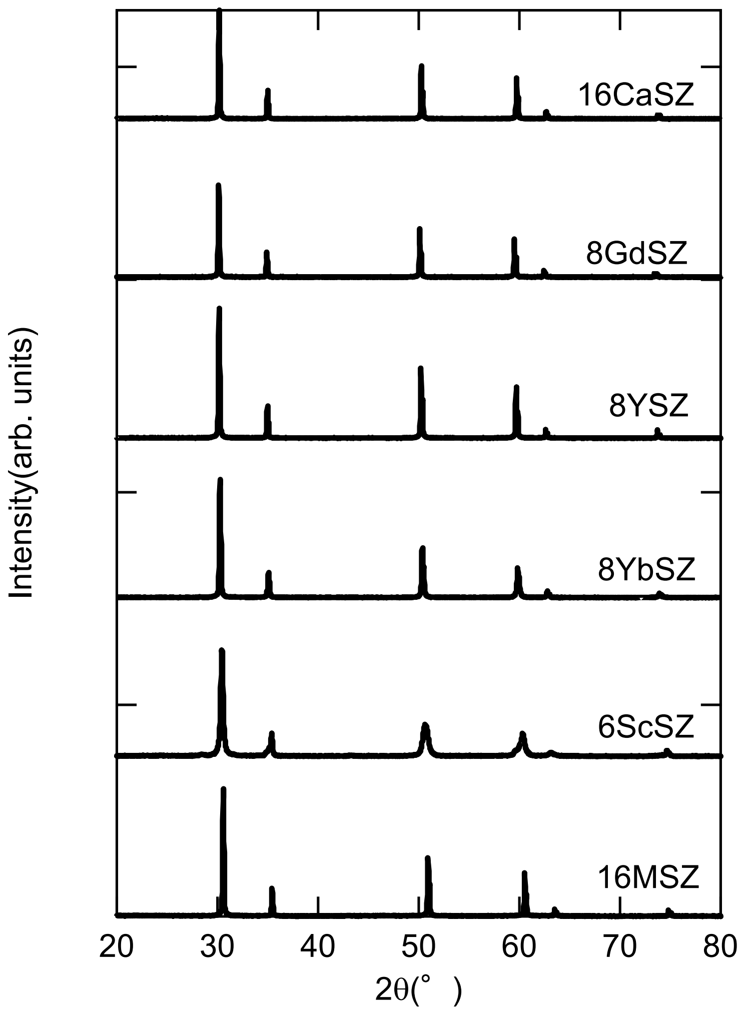

Supplementary Figure 6| XRD patterns of several stabilized zirconia samples with different dopant ions. The 16CaSZ, 8CdSZ, 8YSZ, 8YbSZ, and 16MSZ samples exhibit a cubic structure, and the 6ScSZ sample possesses a tetragonal structure.
